# Transcriptome of rat subcortical white matter and spinal cord after spinal injury and cortical stimulation

**DOI:** 10.1038/s41597-021-00953-4

**Published:** 2021-07-15

**Authors:** Bethany R. Kondiles, Haichao Wei, Lesley S. Chaboub, Philip J. Horner, Jia Qian Wu, Steve I. Perlmutter

**Affiliations:** 1 Department of Physiology and Biophysics, University of Washington, Seattle, WA USA; 2 Center for Neuroregeneration, Department of Neurosurgery, Houston Methodist Research Institute, Houston, TX USA; 3 The Vivian L. Smith Department of Neurosurgery, McGovern Medical School, The University of Texas Health Science Center at Houston, Houston, TX USA; 4 Center for Stem Cell and Regenerative Medicine, UT Brown Foundation Institute of Molecular Medicine, Houston, TX USA; 5 MD Anderson Cancer Center UTHealth Graduate School of Biomedical Sciences, Houston, TX USA

**Keywords:** Spinal cord injury, Transcriptomics

## Abstract

Spinal cord injury disrupts ascending and descending neural signals causing sensory and motor dysfunction. Neuromodulation with electrical stimulation is used in both clinical and research settings to induce neural plasticity and improve functional recovery following spinal trauma. However, the mechanisms by which electrical stimulation affects recovery remain unclear. In this study we examined the effects of cortical electrical stimulation following injury on transcription at several levels of the central nervous system. We performed a unilateral, incomplete cervical spinal contusion injury in rats and delivered stimulation for one week to the contralesional motor cortex to activate the corticospinal tract and other pathways. RNA was purified from bilateral subcortical white matter and 3 levels of the spinal cord. Here we provide the complete data set in the hope that it will be useful for researchers studying electrical stimulation as a therapy to improve recovery from the deficits associated with spinal cord injury.

## Abstract (summary)

**Table:** 

Measurement(s)	RNA
Technology Type(s)	RNA sequencing
Factor Type(s)	motor cortex electrical stimulation
Sample Characteristic - Organism	Rattus norvegicus

Machine-accessible metadata file describing the reported data: 10.6084/m9.figshare.14129012

## Background & Summary

Spinal cord injury (SCI) disrupts motor and sensory signaling below the lesion and negatively impacts the quality of life of affected individuals. Electrical stimulation is an intervention used to modulate the excitability of neurons, induce neural plasticity, and promote functional recovery after damage to the central nervous system (CNS). After SCI, direct stimulation of the spinal cord^1,2^ and brain^3,4^ can aid functional recovery. Nonetheless, little is known regarding the mechanisms of stimulation-induced recovery, which impedes efforts to more selectively target stimulation and design more effective protocols to advance further development of the therapy. One way to understand the effects of neuromodulation is to examine its effects on the transcriptional profile of the CNS.

Studies that describe transcriptional profiles following sub-chronic and chronic SCI^5^ identify potentially important pathways to target for therapeutic strategies. For example, large scale transcriptomic approaches can help identify gene networks involved in preventing or encouraging regeneration (reviewed^6^). Other studies have examined the effects of electrical stimulation on transcription in CNS neurons, such as cultured dorsal root ganglion cells^7^ and hippocampal neurons in freely moving rats^8^. One study endeavored to detail the effects of a brief stimulation of the brain stem on transcription after thoracic SCI^9^. However, to our knowledge a dataset of the effects of electrical stimulation on gene and network expression in a SCI model has not been reported. To help fill this gap, we examined the effects of cortical stimulation in rats with incomplete SCI on the transcriptional profile of the CNS tissue.

**Fig. 1:**
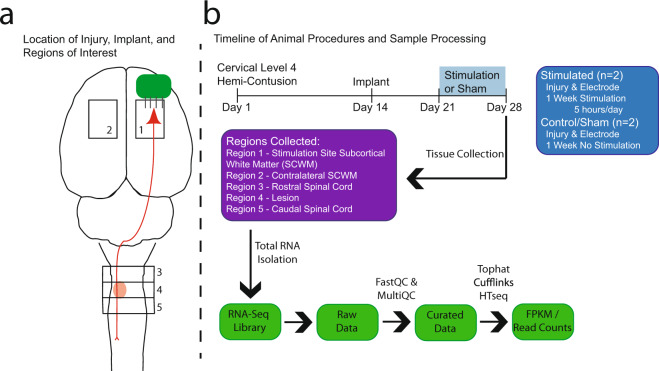
Experimental Design and Timeline. (**a**) Schematic detailing location of electrodes implanted into motor cortex to activate corticospinal neurons (illustrated in red). Hemi-contusion injuries at cervical level 4 disrupted the function of the CST and other pathways (orange oval). Regions 1–5 were isolated for RNA purification. (**b**) Animals first sustained a unilateral cervical injury, then were implanted with stimulating electrodes 14 days later. Stimulation lasted for one week in two animals; two control animals received no stimulation. Following RNA extraction, purification, and sequencing, the workflow included quality control.

Adult female rats were given a unilateral, incomplete cervical injury and the contralesional motor cortex was stimulated electrically through an implanted electrode array for one week. Tissue from 5 regions (Fig. 1a) was processed for total RNA to determine what transcriptional changes occurred in response to the stimulation. Stimulating the motor cortex activates the corticospinal tract (CST) and other corticofugal pathways, cortico-cortical connections, spinal neurons, and subsequent sensory and propriospinal feedback pathways (reviewed by^10^). Stimulation of the motor cortex has been shown to induce morphological and functional changes following injury^3,4,11^. In addition, manipulating levels of neural activity can affect glial populations^12,13^.

We are providing these data without interpretation, as the small sample size prevents drawing strong conclusions. It is our hope that a greater understanding of the transcriptional profile of the CNS in response to electrical stimulation after injury will be a resource for other researchers attempting to identify gene targets for new treatments for SCI. Importantly, the present study only used one frequency of stimulation, although different frequencies and patterns of stimulation have unique effects on functional outcomes following SCI^4^ and transcription of *in vitro* neural cultures^7^. In the future, it will be imperative to learn how transcriptional changes are related to the morphological, synaptic, and behavioral effects^14–16^ produced by different patterns of electrical stimulation. Here, we sought to determine if there is an early effect of stimulation on gene expression in several CNS regions after SCI, without considering if the effects lead to changes in neural circuitry or behavior.

## Methods

### Animals

All animal procedures were approved by the University of Washington Institutional Animal Care and Use Committee. Four adult female Long-Evans rats weighing 275–350 grams were kept on a 12 hour light/dark cycle with *ad libitum* access to food and water. Each animal’s dominant forelimb was established via their performance on a pellet retrieval task^17^ prior to injury.

### Surgical procedures

Figure 1b shows the experimental timeline and design. Animals were anesthetized with ketamine/xylazine (50 mg/kg and 1 mg/kg, respectively). Local anesthetic (lidocaine at 1 mg/kg and bupivacaine HCl at 1 mg/kg) was administered to the skin overlying the dorsal aspect of the cervical spinal column. The dorsal surface of the spinal cord on the side of the dominant forelimb was exposed with hemi-laminectomy and opening of the dura under aseptic surgical conditions. Animals sustained a cervical level 4 (C4) hemi-contusion injury using a third generation of the Ohio State Impact Device calibrated to cause a displacement of 0.7 mm. This incomplete injury, used frequently in our laboratory, damages the local gray matter and partially disrupts the CST and lateral white matter tracts, and produces a moderate deficit characterized by a flexor bias and weakness in the ipsilateral forelimb^18^. The injury site was covered with gel foam, the muscles were closed in layers with absorbable suture, and the skin was closed with nylon suture. Subcutaneous injections of lactated Ringers and buprenorphine slow release (1.2 mg/kg) were given, and the animals woke up in a warmed recovery cage. Bladders were manually expressed twice daily until the return of normal micturition. Animals were administered Baytril antibiotic in their water bottles (25 mg/kg) for 14 days. All animals exhibited the expected deficit of weight bearing and use of the affected forelimb.

After two weeks of recovery, a sub-cutaneous injection of dexamethasone (0.2 mg/kg) was given to prevent brain swelling, and 24 hours later animals were anesthetized with 3% isoflurane in oxygen. Under aseptic surgical conditions the skull was exposed, and a craniotomy was performed over the contralesional caudal forelimb motor area (Fig. 1a). Approximate coordinates of the craniotomy were 5 mm anterior to 1 mm posterior of bregma, and 1–4 mm lateral to the midline. A stimulating electrode array (described below) was implanted into cortex with the tips of the tungsten wires penetrating 1 mm. The craniotomy was filled with gel foam and the electrode array secured with dental acrylic to the skull, creating a cap which sealed the incision. Animals were administered analgesic and antibiotic drugs as described above.

### Stimulation

Stimulating electrodes consisted of a 2 × 4 array of polytetrafluoroethylene-insulated, tungsten wires (0.002” bare, 0.004” coated), with impedances less than 30 kOhm. The ends of the wire were deinsulated, trimmed, and cut at a 45° angle so that a sharp, stripped (<0.5 mm) tip resulted. The array was mounted onto a printed circuit board with a connector wired to the electrodes. One week after implantation, the efficacy of each intracortical wire to activate CST neurons was determined by measuring the threshold current for evoking forelimb movements^10^ on the lesioned side. For this test, biphasic pulses at 10 Hz at currents ranging from 50–250 µAmps were delivered to each wire. A skull screw far from motor cortex served as the return electrode. For animals assigned to the stimulation group, the electrode that elicited the clearest forelimb movement at the lowest threshold was chosen as the stimulation channel for that animal for the remainder of the study. All animals were kept in a behavioral arena for 5 hours each day. Animals in the stimulation group (n = 2) were connected by a cable to the Neurochip, a custom-built, autonomous brain-computer interface that delivered biphasic pulses to the chosen stimulation channel at an average of 10 Hz for cycles of 5 minutes on/2 minutes off at 80% of movement threshold, which activates neurons without causing large contractions^1,3^. Control animals (n = 2) received the same SCI and implant and were treated the same as electrically stimulated animals, including connection to a sham Neurochip, but did not receive stimulation. For consistency and to avoid circadian effects, stimulation was always begun in the morning. Stimulation was administered for 5 days, starting 3 weeks after injury. Personnel were not blinded to stimulation condition, as they did not make any assessments that could be biased by a knowledge of treatment condition.

### Tissue collection

After the stimulation period ended, animals were anesthetized with a lethal injection of Beuthanasia-D and perfused transcardially with ice cold phosphate buffered saline. Spinal cords and brains were removed, and five designated regions (Fig. 1a) were dissected out and snap frozen in liquid nitrogen. Region 1 was an approximately 5 mm^3^ block of subcortical white matter directly below the implantation/stimulation site; this region includes axons, and glia of many corticofugal and corticopetal pathways. Region 2 was the same region on the contralateral side, which served as an internal control since it did not receive any direct stimulation. The cortical dissection isolated the white matter only, discarding the cortex. Region 3 was spinal cord rostral to the injury (~C2-C4). Region 4 was the lesion epicenter (C4). Region 5 was the spinal cord caudal to the injury (~C5–C7). The spinal cord regions included both gray and white matter, neuron cell bodies and processes, and glia. At the post-injury timepoint when the tissue was sampled, the white matter included both injured and spared axons of many descending and ascending tracts, including retracting and sprouting fibers. Tissue was stored at −80 °C until RNA isolation and purification.

### RNA extraction, library preparation, and sequencing

Tissue was homogenized and complete RNA was isolated using an RNAeasy Mini Kit (Qiagen) according to the manufacturer’s protocol. TruSeq Total RNA sequencing with ribosomal depletion was conducted by the Weill Cornell Epigenomics Core. The cDNA-libraries were sequenced by Illumina Hiseq. 2500 sequencer (paired-end, 2 × 51 bp). Sequencing was done together to control for batch effects, without any negative or spike-in controls.

### RNA-seq data analysis

The quality of the reads was verified using FastQC (https://www.bioinformatics.babraham.ac.uk/projects/fastqc/). The assessment results were summarized and visualized using MultiQC 1.6^19^. The rat reference genome Rnor6 was downloaded from Ensembl (ftp://ftp.ncbi.nlm.nih.gov/genomes/all/GCF_000001895.5_Rnor_6.0/). The annotated file for the rat genome was taken from our previous studies^5^. Read mapping, transcript assembly, and expression estimation were performed as described in our previous publications^5,20^. The 51-bp paired-end reads were aligned to the reference genome using TopHat v2.1.0 using default parameters^21,22^. Quality control and mapping rate metrics are listed in Table 1. Fragments per kilobase of transcript per million mapped reads (FPKM) values were obtained for genes and transcripts using Cufflinks v2.2.1^21,23^. To avoid inflation of ratios with small denominators, FPKM values < 0.1 were rounded up to a value of 0.1^24^. Next, we removed genes with FPKM <1 across all samples, as these genes were not expressed or were expressed at very low levels in all samples. A total of 15,210 protein coding genes and long non-coding RNAs were used for further analysis. The distribution of genes expressed in each sample was analyzed by ggplot2. A principal component analysis (PCA) was performed on log2-transformed FPKM values using the R function “prcomp” and the setting “scale = TRUE”. The coefficient of variation (CV) of the transformed FPKM values across samples was calculated for each gene and the CVs were ranked from largest to smallest. The most variable genes, taken to be the 10% with the largest CVs, were included in the PCA analysis. Three-dimensional PCA plots were designed by R package “pca3d’. Integrative Genomics Viewer (IGV) was used to visualize gene expression levels across regions and animals^25^.

**Table 1:** The summary of RNA-Seq statistics.

Sample	Read1 No.	Read1 Length	Read1 Mapping Rate	Read2 Mapping Rate	RIN Scores
Control1_region1	53,792,745	51	92.70%	88.60%	9.6
Control1_region2	56,315,463	51	93.60%	89.70%	9.6
Control1_region3	59,005,995	51	92.90%	88.70%	9.3
Control1_region4	59,208,539	51	92.30%	88.40%	9.5
Control1_region5	52,613,415	51	93.00%	88.50%	10
Control2_region1	64,115,161	51	92.70%	88.80%	9.3
Control2_region2	56,948,286	51	94.00%	89.60%	9
Control2_region3	60,970,792	51	91.80%	87.60%	8.9
Control2_region4	53,463,239	51	93.50%	89.50%	9.2
Control2_region5	56,028,200	51	90.40%	86.60%	9.6
Treatment1_region1	61,564,749	51	93.10%	88.70%	8.6
Treatment1_region2	60,636,797	51	93.50%	89.30%	9
Treatment1_region3	55,606,216	51	90.60%	86.90%	9.5
Treatment1_region4	57,935,339	51	93.30%	89.00%	9.8
Treatment1_region5	58,486,827	51	93.00%	88.90%	9.6
Treatment2_region1	59,223,921	51	90.70%	86.80%	9.6
Treatment2_region2	56,893,902	51	92.60%	88.40%	9.6
Treatment2_region3	56,653,588	51	92.40%	88.00%	9.5
Treatment2_region4	59,414,002	51	93.10%	89.30%	9.2
Treatment2_region5	60,840,758	51	92.10%	88.30%	9.8

The mapping rate of read1 or read2 was ranked from 86.6% to 94.0%. The RIN scores for all samples of the RNA-Seq library were more than 8.6.

## Data Records

The raw RNA-seq data were deposited in NCBI Gene Expression Omnibus (GEO) with accession number GSE155610^26^. This GEO project includes raw data in FastQ format and FPKM values for all samples.

## Technical Validation

### RNA Quality

The purity and integrity of the total RNA were assessed with the Agilent Bioanalyzer 2100. The RNA samples with RIN (RNA integrity numbers)^27^ >8.6 were used to prepare RNA-seq libraries. Table 1 shows the RNA quality values in this study.

### RNA-seq quality validation

**Fig. 2:**
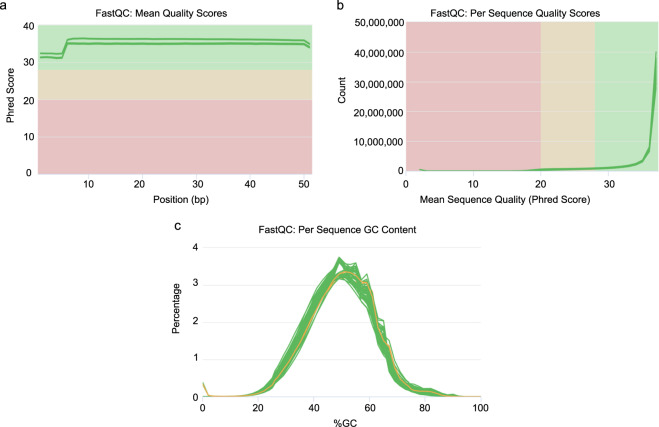
MultiQC summary plot of FastQC quality assessment for the raw FASTQ sequence data in all samples. (**a**) The distribution of the mean quality value per base in sequencing reads. (**b**) The distribution of the mean quality scores per sequence (x-axis). (**c**) The distribution of guanine-cytosine (GC) content for all sequences, shown as the percentage of reads with specific GC values from 1–100%.

**Fig. 3:**
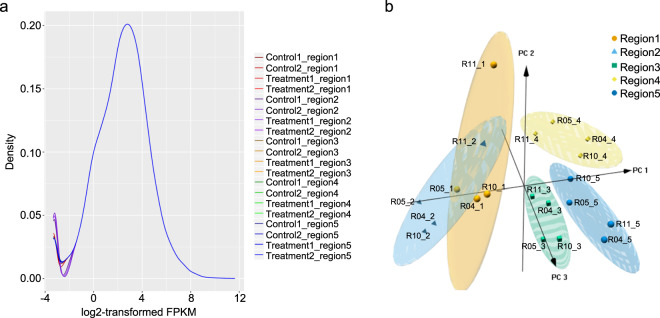
The identification of RNA-Seq data in all samples. (**a**) The distribution of gene expression values of all samples. The X-axis represents log2-transformed quantile normalized FPKM values. (**b**) 3D plot of PCA using the 10% of genes with the largest CVs of transformed FPKM values across all samples. Grouping of correlated samples is indicated by color-shaded ovals. Data points are labeled by rat number and region number. For example, R04_1 is region 1 from rat 04. Rats R04 and R05 were unstimulated control animals; rats R10 and R11 received stimulation.

**Fig. 4:**
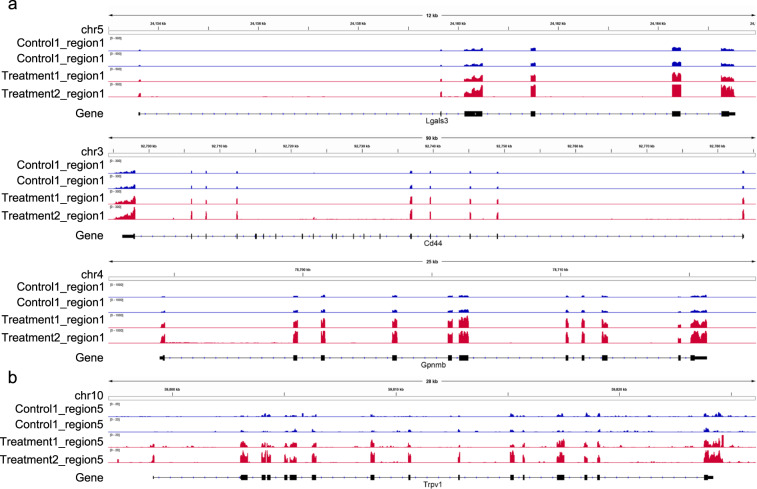
Genome browser views displaying gene expression levels. Gene expressing tracks displaying *Lgals3*, *Cd44*, and *Gpnmb* in Region 1 (**a**) and *Trpv1* in Region 5 (**b**) of two control and two treatment samples.

We applied FastQC to evaluate the mean per-base quality scores, per sequence quality scores and per sequence GC content. The per base quality scores were higher than phred quality 30^28^ and most sequences had a quality over 20 (Fig. 2a,b). The GC contents of the samples showed a similar normal distribution^29^, indicating the samples were free of contamination (Fig. 2c). These results showed the RNA-seq reads had high quality. The experiment yielded 20 samples with a total of 1,159 million reads. After mapping reads, 86.6–94% of the reads were mapped to the Rat genome (Table 1). The gene expression had a similar distribution across all samples (Fig. 3a). The genes were clustered by region in three-dimensional PCA plots (Fig. 3b). Gene expression patterns in Regions 1 and 2 were distinct from the other 3 regions. In order to assess the quality of the RNA-seq libraries, the transcripts of known highly expressed genes were examined. Compared with unstimulated animals, *Lgals3*, *Cd44* and *Gpnmb* in region 1 (Fig. 4a) and *Trpv1* in region 5 (Fig. 4b) were more highly expressed after stimulation.

## Data Availability

All analyses were performed using open sources software tools with default parameters (please see Methods).
